# Help Comes from Understanding: The Positive Effect of Communication Visibility on Employee Helping Behavior

**DOI:** 10.3390/ijerph17145022

**Published:** 2020-07-13

**Authors:** Liang Liang, Guyang Tian, Xue Zhang, Yezhuang Tian

**Affiliations:** School of Management, Harbin Institute of Technology, Harbin 150001, Heilongjiang, China; 18b910052@stu.hit.edu.cn (L.L.); tianguyang07@163.com (G.T.); 15b910027@hit.edu.cn (X.Z.)

**Keywords:** communication visibility, helping behavior, trust in coworkers, proactive personality

## Abstract

Extant research focuses on the antecedents of employee helping behavior, but the role of social technologies in enhancing employee helping behavior remains understudied. The purpose of our research is to investigate the relationship between communication visibility and employee helping behavior. Drawing on both communication visibility theory and social cognitive theory, we propose that the association between communication visibility and helping behavior is mediated by employee psychological state assessed by a cognitive state variable: trust in coworkers. Further, we also propose that proactive personality moderates the positive effect of trust in coworkers on employee helping behavior. We examined our hypothesized relationships using 149 employees collected in a field experiment in China. As hypothesized, we find that trust in coworkers mediates the relationship between communication visibility and helping behavior. Moreover, proactive personality strengthens the effectiveness of communication visibility. We discuss the implications of our findings for future research and practice.

## 1. Introduction

Nowadays, enterprise social media (ESM), such as Yammer and Jive [1], are widely used in increasing numbers of enterprises. The prevalence and practical value of ESM have attracted the attention of scholars [2,3]. ESM platforms allow employees to obtain information in ways that are hard to be realized via traditional media [2,4]. In particular, these platforms mean that employees know more about the content of their coworkers’ communication information and networks [5], which is known as “communication visibility”. Communication visibility can affect how employees access information, acquire resources, and improve performance [6]. For this change in employees’ ways of working, it is crucial to consider the potential outcomes of communication visibility for the sustainable development of enterprises in today’s hyper-competitive environment.

Communication visibility captures the tendency for employees to utilize technology in ways that make any user able to easily see what others say and to whom they say it [7,8,9]. For instance, an employee can see the communication activities among their coworkers even though he is the third party to the communication and not directly involved [7]. Studies find that communication visibility is associated with employee knowledge sharing and innovation by nurturing message transparency and network translucence climate [5,7]. Sun et al. [2] support the contention that communication visibility is positively related to employee creative performance. All these studies reveal the relationship between communication visibility and different types of performance. However, how helping behavior, as an important aspect of performance, [10] influences communication visibility has remained unclear. Helping behavior refers to the voluntary assistance of others in accomplishing their goals or preventing the occurrence of problems [11,12], which is also regarded as a type of organizational citizenship behavior [12,13]. Ter Hoeven et al. [14] suggest that future studies should examine the effect of visibility on organizational citizenship behavior. Thus, it is valuable to explore why, how, and when communication visibility facilitates employee helping behavior.

Our research introduces social cognitive theory to explain how communication visibility influences employee helping behavior. Social cognitive theory highlights that environmental information affects individual cognition and behavior [15,16]. Deutsch [17] considers trust to be a response to environmental stimuli that can influence an individual’s cognition and behavior. Tan and Lim [18] define trust in coworkers as “the willingness of a person to be vulnerable to the actions of coworkers whose behavior and actions that person cannot control” (p. 46). As such, we select “trust in coworkers” as the cognitive psychological state variable in our study. According to communication visibility theory, communication visibility creates an environment that can bring employees together and spark more conversations among coworkers [7]. These characteristics may help employees know more about coworkers’ competences, skills, and their similar preferences, which can be conducive to employees’ trust in their coworkers. Several studies highlight a close association between trust and helping behavior [13,19,20,21]. Therefore, we expect that trust in coworkers may mediate the association between communication visibility and employee helping behavior.

We further investigate whether the link between communication visibility and helping behavior is moderated by employee personality. Communication visibility nurtures an environment that may result in trust in coworkers, but this does not mean that employees engage in helping behavior. Social cognitive theory proposes that both personal factors and environmental factors affect individual behavior [20,22]. As a personal factor, proactive personality refers to a relatively stable behavioral tendency to identify opportunities to change things at work and to act on the impulses [23,24,25]. Previous research has found that proactive personality is a personal factor that facilitates helping behavior [24,26]. Employees with a proactive personality are more likely to make positive inferences about their work environment [27]. Drawing on the existing literature, we expect that proactive personality moderates the relationship between trust in coworkers and helping behavior.

**Figure 1:**
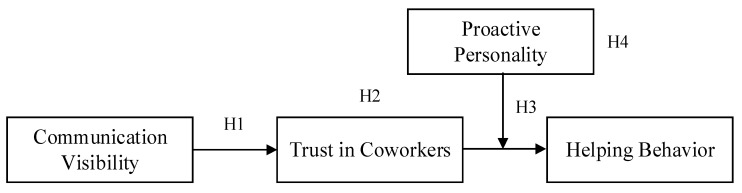
Conceptual model for communication visibility and helping behavior.

In summary, our study mainly concentrates on two critical aspects. First, relatively less is known about how communication visibility influences employee helping behavior. We explore the indirect effect of communication visibility on employee helping behavior through trust in coworkers from the perspective of cognitive psychological state. Second, we introduce the proactive personality variable and test the moderating effect of proactive personality on the influence of trust in workers on helping behavior. Thus, the boundary condition under which communication visibility functions is explored in our research. Figure 1 represents the relations between the variables discussed above.

## 2. Theoretical Background and Hypotheses

### 2.1. Communication Visibility and Trust in Coworkers

Trust is required for coworkers to work together effectively [28]. Due to the critical role of trust in organizations, trust has been linked to various positive workplace outcomes [29]. Researchers have found that trust in coworkers can increase proactive behavior in the workplace [30]. The current study suggests that communication visibility is likely to increase trust in coworkers based on communication visibility theory.

There are two reasons to suggest a positive relationship between communication visibility and trust in coworkers. On the one hand, communication visibility theory states that communication visibility enables employees to know more about their coworkers’ knowledge, as well as the content of others’ messages [5]. Thus, employees can identify experts in relevant fields and acquire work-related knowledge from their coworkers [2], which can save their time and efforts when seeking knowledge. Therefore, employees may be easier to form trust regarding coworkers’ competence. Mayer et al. [31] suggest that competence trust is an essential component of trust in coworkers. On the other hand, communication visibility theory also argues that communication visibility includes network translucence [5]. The apparent social networks can make employees develop an awareness of coworkers’ connections [32], allowing employees to quickly find common ground [33], similar interests and visions, which may result in trust among coworkers [1]. Levin [34] indicates that although frequent interactions may not always build trust, bringing employees together may spur conversations that can signal one’s benevolence. Communication visibility can increase interactions, as well as conversations among employees, even though they are not familiar with their work. Besides, coworkers are more responsible for what they say in such a visible environment. According to the logic of Levin [34], communication visibility may be conducive to benevolence-based trust, which is also an important component of trust in coworkers. Thus, compared to a traditional context where employees work without ESM and with low levels of communication visibility, employees may get to know their coworkers and build trust relationships more quickly when they work in an environment with a high level of communication visibility. We thus hypothesize:

**Hypothesis** **1** **(H1).**  *Communication visibility will be positively related to trust in coworkers.*

### 2.2. The Mediating Effect of Trust in Coworkers

Early research shows that trust in coworkers is related to more proactive behavior in the workplace [30]. Employee helping behavior, as a kind of proactive behavior, refers to the voluntary assistance of others in accomplishing goals and preventing the occurrence of problems [35]. We propose that trust in coworkers mediates the relationship between communication visibility and employee helping behavior.

The social cognitive theory posits that environmental information may indirectly influence an individual’s cognition and behavior [15]. It states that an individual will take actions based on personal cognitions in a social environment [16]. Employees will evaluate the costs and benefits of their actions before engaging in an act [36]. Trust means individuals believe that the other party will behave in a dependable manner and will not take advantage of the environment [37]. When employees trust their coworkers, they accept costs associated with maintaining these relationships more easily. This is because they feel more confident that their investments in the relationship will reap adequate returns [38]. Therefore, employees are willing to engage in more helping behavior. Several studies have highlighted a close relationship between trust and helping behavior [20]. Combining communication visibility theory with social cognitive theory, we argue that employees are more likely to engage in helping behavior when employees trust their coworkers as a result of communication visibility. Taking the above arguments and evidence together, we offer the following hypothesis:

**Hypothesis** **2** **(H2).**  *Communication visibility will have a positive and indirect relationship with employee helping behavior through trust in coworkers.*

### 2.3. The Moderation of Proactive Personality

As mentioned previously, the social cognitive theory emphasizes that both personal factors and environmental factors affect individual behavior [20]. Therefore, we should consider personal personality in our research. Previous research has found that proactive personality may facilitate helping behavior [24] and contribute to building relationships with coworkers [39]. Thus, we infer that the relationship between employees’ trust in coworkers and helping behavior depends on the proactive personality of employees.

Proactive employees struggle to create a better work environment and generate opportunities for themselves. Research on proactive personality has suggested that employees vary in their expectations regarding their ability to change the environment [23,40]. We propose that when employees trust their coworkers, different personalities may have different evaluations of their coworkers’ behavior. This may further influence whether employees will help their coworkers. Proactive employees may believe that their work environment is benevolent and hold positive expectations of others [41]. They will also be more willing to exhibit pro-social behavior to make the workplace more enjoyable [42]. Therefore, when employees with a high level of proactive personality trust their coworkers, they will be more likely to believe that their coworkers will repay their efforts when they help their coworkers. In contrast, employees with a low level of proactive personality may fail to change the work environment and pay less attention to gaining work resources [43]. Therefore, even though they trust their coworkers, they will be less likely to evaluate the work environment proactively, which may not be conducive to employee helping behavior. Based on the arguments above, we hypothesize that:

**Hypothesis** **3** **(H3).**  *Proactive personality positively moderates the relationship between trust in coworkers and helping behavior, such that this relationship will be stronger when proactive personality is higher rather than lower.*

### 2.4. The Moderated Mediation Hypothesis

In summary, we suggest that proactive personality moderates the indirect effect of communication visibility on employee helping behavior through trust in coworkers. In other words, for proactive employees, the indirect effect of communication visibility on helping behavior may be stronger. However, the effect of communication visibility on helping behavior through trust in coworkers will be weaker for employees with a low level of proactive personality. As such, this moderated mediation model clarifies why (trust in coworkers) and when (a high level of proactive personality) communication visibility results in helping behavior. To test this moderated mediation model, we postulate the following hypothesis:

**Hypothesis** **4** **(H4).**  *Proactive personality moderates the indirect effect of communication visibility on helping behavior via trust in coworkers, such that the indirect effect is stronger when proactive personality is higher than lower.*

## 3. Method

### 3.1. Participants and Procedure

We collected data by conducting a field experiment at CBM (a pseudonym for anonymity), a large private-service company with more than 1000 employees in the Northeast of China. We have traced this company for seven years and visited the firm and conducted in-depth interviews with the CEO, as well as its employees, several times to understand the research setting. Before the field study, all employees at CBM had been using Enterprise WeChat, one of the most widely used ESM platforms in China [44]. At first, the company did not make all functions available to employees. Employees could use it only for a simple collaboration, such as sending files to their coworkers. However, this was often person-to-person, and employees could not share their ideas freely via Enterprise WeChat. In December of 2016, the enterprise decided to introduce and implement a new function of Enterprise WeChat called “DB” (short for “Dissipation Bar”). It encouraged employees to freely communicate via “DB”. Employees could post and share their knowledge, ideas, questions, or advice related to their work, with their posts being seen by the whole company. After someone posted via “DB”, others could respond to the content provided by the poster (e.g., comments, likes, or dislikes). Therefore, after the introduction of new functions, employees knew more about “who knows whom” and “who knows what”. That is to say, employees had an environment with a much higher level of communication visibility than before.

Fortunately for this study, the enterprise did not open this new function to all employees. The team responsible for the implementation of “DB” selected five departments (e.g., Operating Department) at random to participate in “DB” initially and other departments still used the old simple version of Enterprise WeChat. In March of 2017, three months after “DB” was implemented in five departments, we contacted 130 participants from departments where “DB” had not been implemented (control condition) and 150 participants from those departments where “DB” had been implemented (treatment condition), at random. In our initial contact with the participants, a general overview of the research was provided (e.g., organizational behavior research). However, we did not disclose the specific research hypotheses. All participants were assured that their responses would remain confidential. Besides, all participants gave their informed consent for inclusion before they voluntarily participated in the study. The study was conducted in accordance with the Declaration of Helsinki of 1975. Participants from both groups were asked to complete measures of trust in coworkers, helping behavior, proactive personality, and demographics. Specifically, participants from the control group were asked to answer questionnaires regarding their feelings when using Enterprise WeChat. In contrast, participants from the treatment condition were asked to answer questionnaires related to their feelings when using the new “DB” function over the previous three months. In total, 280 employees participated in our survey. After eliminating surveys with missing responses, we had 149 valid questionnaires, yielding an effective response rate of 53.21%. Especially, there were 94 participants in the treatment condition and 55 in the control condition. On average, the participants included 55.70% males and their age ranges were 20–25 (12.75%), 26–35 (69.80%), and over 36 years (17.45%). Their primary education was undergraduate degree (64.43%). Table 1 shows more demographic characteristics of these participants.

### 3.2. Measurements

All constructs were multiple-item scales adapted from existing scales and were answered using five-point Likert scales, ranging from: (1) “strongly disagree” to (5) “strongly agree”. Questionnaires included three constructs, namely trust in coworkers, helping behavior, proactive personality. Demographic variables were also included.

Allowing for the characteristics of each group, we treated communication visibility as a binary variable. Samples from the treatment group were recorded as 1, and samples from the control group were recorded as 0.

Trust in coworkers was assessed using the six-item measurement adapted from Mc Allister [45], Mayer and Davis [46] and Gould-Williams [47]. A sample item was “I trust in my coworkers’ ability in my work”. The Cronbach’s alpha for the trust in coworkers scale was 0.87.

Seven items from Podsakoff, Ahearne, and MacKenzie’s [48] were used to measure helping behavior. A sample item was “Willingly share their expertise with other members of the crew”. The Cronbach’s alpha for the helping behavior scale was 0.85.

To measure proactive personality, we used a well-established scale by Seibert et al. [49], which includes ten items (α = 0.88). A sample item was “Wherever I have been, I have been a powerful force for constructive change”. The Cronbach’s alpha for the proactive personality scale was 0.88.

A few participants’ demographic variables were controlled, including gender, age, work tenure, and education. Previous studies have shown that these variables are related to helping behavior [13,50].

### 3.3. Analytic Strategy

Before data analysis, all the variables used in the interaction terms were mean-centered. Then, confirmatory factor analysis was performed to assess the distinctiveness of these variables. The model (Figure 1) of the hypothesized direct and indirect effects was tested by hierarchical multiple regression analysis using SPSS Statistics 23.0 for Windows (IBM Corp, Armonk, NY, USA). For the indirect effects, bootstrapping was used to test the mediating effects [51].

## 4. Results

To statistically test the demographic difference between the two groups, we conducted t-tests. The results showed that all *p* values of *t*-tests for demographic variables were greater than 0.05 (*p* = 0.83 for gender, 0.70 for age, 0.56 for education, 0.74 for work tenure), which indicated that there was no significant difference among the baseline characteristics of the two groups. Based on this, we analyzed as follows.

### 4.1. Descriptive Statistics

Table 2 presents the means, standard deviations, and correlations of the variables. The results showed that communication visibility was positively correlated with trust in coworkers (r = 0.25, *p* < 0.01) and helping behavior (r = 0.16, *p* < 0.05). Besides, employee trust in coworkers was also positive to employee helping behavior (r = 0.21, *p* < 0.01).

### 4.2. Confirmatory Factor Analysis

To evaluate the construct validity of variables, we conducted confirmatory factor analyses (CFA). The results demonstrated that the hypothesized three-factor model was a good fit with the data (χ^2^ = 356.15, df = 213, χ^2^/df = 1.67, CFI = 0.92, TLI = 0.90, RMSEA = 0.07), hypothesized three-factor model fit the data better than two-factor and one-factor models (see Table 3).

### 4.3. Hypotheses Testing

Data were analyzed with hierarchical regression analysis using SPSS Statistics Version 23.0. First, we conducted a t-test to analyze the relationship between communication visibility and trust in coworkers. The results showed that participants in the communication visibility condition (*N* = 94, M = 4.14, SD = 0.40) were more likely to trust in coworkers than those in the control condition (*N* = 55, M = 3.92, SD = 0.43) and the difference was significant (t = 3.10, *p* < 0.01). Therefore, Hypothesis 1 was supported. Besides, Table 4 shows the results of the hierarchical regression analysis. Consistent with the predictions, communication visibility was positively related to trust in coworkers (b = 0.22, *p* < 0.001; Model 2), which also supported Hypothesis 1.

Second, Hypothesis 2, which proposes that trust in coworkers can mediate the relationship between communication visibility and helping behavior, was tested. Communication visibility was positively related to trust in coworkers (b = 0.22, *p* < 0.001; Model 2) and helping behavior (b = 0.19, *p* < 0.05; Model 4). When trust in coworkers as the mediator variable was added into the model, trust in coworkers had a significant positive impact on helping behavior (b = 0.22, *p* < 0.05; Model 5). However, the effect of communication visibility on helping behavior became non-significant (b = 0.14, n.s.; Model 5).

We further tested the indirect effect of communication visibility on helping behavior. According to Preacher et al. [51], bootstrapping was used by drawing 5000 random samples to test the significance of the mediation hypothesis (PROCESS Model 4). The results showed that communication visibility had an indirect effect on helping behavior through employee trust in coworkers (b = 0.05, SE = 0.02, 95% CI [0.01, 0.09]). The 95% confidence interval excluded zero, which indicated that trust in coworkers mediated the relationship between communication visibility and helping behavior. These results supported Hypothesis 2.

**Figure 2:**
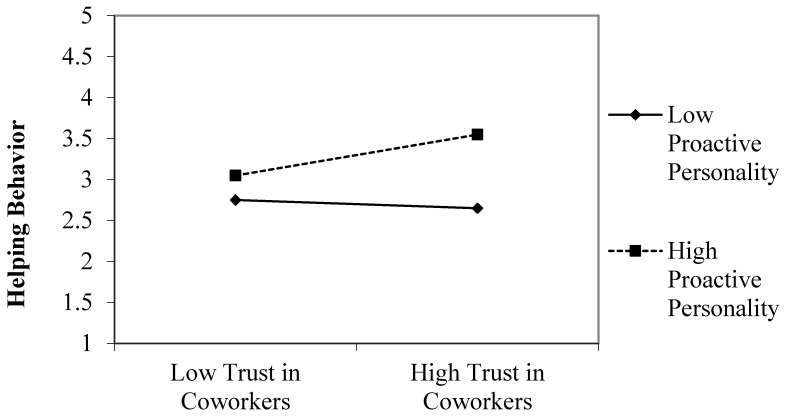
The interactive effect of trust in coworkers and proactive personality on helping behavior.

Table 4 also shows the results of the moderated hierarchical regression analysis for Hypothesis 3. The interaction of trust in coworkers and proactive personality was significant and positive for helping behavior (b = 0.37, *p* < 0.05; Model 8). Figure 2 illustrates the results of a simple slope analysis. As shown in Figure 2, trust in coworkers had a stronger positive relationship with helping behavior when proactive personality was high (1 SD above the mean) (simple slope = 0.25, *p* < 0.001). However, another simple slope analysis showed that trust in coworkers did not have a significant relationship with employee helping behavior when proactive personality was low (1 SD below the mean) (simple slope = −0.05, n.s.). Therefore, Hypothesis 3 was supported.

Finally, we used SPSS PROCESS Model 14 to examine the moderated mediation hypotheses (i.e., Hypothesis 4) and the results are shown in Table 5. When proactive personality was high, communication visibility had a significant indirect effect on helping behavior through trust in coworkers (indirect effect = 0.07, SE = 0.03, 95% CI [0.01, 0.15]). When proactive personality was low, however, the mediated model was nonsignificant (indirect effect = −0.02, SE = 0.05, 95% CI [−0.12, 0.06]). The index of moderated mediation was likewise significant (indirect effect = 0.09, SE = 0.06, 95% CI [0.008, 0.228]), providing full support for Hypothesis 4.

## 5. Discussion

In this study, we investigated the relationship between communication visibility and employee helping behavior, drawing on communication visibility theory and social cognitive theory. Based on a sample of 149 employees, our findings support the assumption that trust in coworkers, a cognitive variable, is positively related to communication visibility and helping behavior. Our findings indicate that trust in coworkers mediates the relationship between communication visibility and helping behavior. In addition, our findings also indicate that proactive personality may moderate the process of the effect of communication visibility on helping behavior. This paper makes several contributions. First, it extends the research on communication visibility as antecedents of employee behaviors. Although ter Hoeven et al. [14] suggest that future study should reveal the relationship between visibility and organizational citizenship behavior, the relationship between the two constructs has hardly been explored. Considering helping behavior is a typical type of organizational citizenship behavior, our work provides an accurate and complete understanding of the relationship between communication visibility and employee helping behavior. This research is important since communication visibility may be common in the workplace. The outcomes of communication visibility should be explored in detail to conform to the trend of ESM usage in employees’ work.

Second, the mechanisms underlying communication visibility are still insufficiently studied. We advance the literature by considering trust in coworkers as the psychological mechanism explaining the effect of communication visibility on employee helping behavior. Although, researchers have proposed that trust can increase employee helping behavior. However, prior research has not yet tested it as a mediator linking communication visibility and employee helping behavior. By combining communication visibility theory with social cognitive theory, we reveal the effect of communication visibility on employee cognition and behavior.

Finally, based on communication visibility theory and social cognitive theory, we provide insight regarding how employee proactive personality affects the effect of trust in coworkers caused by communication visibility on helping behavior. Our work is relatively early to expand the boundary of communication visibility research by identifying new conditions relevant to individual personality.

### 5.1. Theoretical Implications

Our research makes several important theoretical implications for the literature on communication visibility and organizational behavior. First, our research enriches the communication visibility theory. We combine communication visibility theory with social cognitive theory to explain the association between communication visibility and employee helping behavior, extending the application and scope of communication visibility theory. Our work echoes the claim that much work is needed to refine communication visibility theory, introduce its scope, and test its predictions in different organizational contexts [7]. Our work extends the understanding of the outcomes of communication visibility and provides a more accurate view of the association between communication visibility and employee helping behavior.

Second, we find that trust in coworkers mediates the effect of communication visibility on helping behavior. In particular, trust in coworkers expands our knowledge of the effects of communication visibility. That is, the work environment created by communication visibility allows employees to know more about their coworkers’ knowledge and preferences, which is conducive to fostering trust in coworkers and helping behavior. We reveal the mechanism of this process according to social cognitive theory: environment information may affect employee helping behavior via their cognition psychological state [16].

Third, the moderating effect of proactive personality also contributes to new knowledge about the boundary of how communication visibility influences helping behavior. Employees with a highly proactive personality may make a positive evaluation of their coworkers, which may reinforce the relationship between trust in coworkers resulting from communication visibility and helping behavior. Compared with previous research, our paper is among the first to shed light on the boundary conditions of communication visibility.

### 5.2. Practical Implications

Our research has practical implications for both managers and employees. First, our study suggests that communication visibility may foster employee helping behavior via increasing trust in coworkers. Although different enterprises introduce different ESM into employee workplace and different ESM have different affordances, research shows that communication visibility is a root affordance [52]. In order to foster employee helping behavior, leaders should consider communication visibility while implementing the ESM in their enterprises and increase employees’ awareness of ESM communication visibility. Leaders can also watch out for the successful use of ESM communication visibility in some enterprises and use them as examples to inspire their employees.

Second, we find that communication visibility may help build employees’ trust in their coworkers, increasing the likelihood that they will help their coworkers. From the perspective of employees, staff members should take advantage of communication visibility in their work environment. Employees should not be worried that their communication information is being seen by their coworkers. Instead, they should communicate with their coworkers more proactively. This would let them know more about coworkers’ knowledge and preferences, which is beneficial for coworkers’ helping behavior.

Third, our results suggest that not all employees with a high level of communication visibility will engage in helping behavior. To increase employee helping behavior, organizations should implement strategies that could develop proactive personality in employees. Especially for those enterprises that are inclined to use ESM, they should not only consider their ESM’s communication visibility but also the positive effects of proactive personality in their technological work environment when they engage in personnel selection.

### 5.3. Limitations and Future Research

This study has several limitations that open new research opportunities for future research. First, it would be better to have a measure of communication visibility that could confirm that employees in the “DB” group with a high level of communication visibility. However, using the approach of Karahanma et al. [53], we reviewed the enterprise objective descriptions of the original version of the ESM and the new “DB” version. We also interviewed managers, as well as employees, to identify the most popular features of the two versions. Three of the authors independently mapped each feature to the list of affordances already identified from the literature (e.g., communication visibility affordance, editability affordance) and found that communication visibility affordance appeared in the “DB” group. We think this finding may result from the fact that not all enterprise social media highlight the communication visibility affordance. For example, the initial version of Enterprise WeChat employed in our study can only allow employees to send files between individuals and employees cannot see communications from their coworkers. Although we have tried to ensure the differences between the two study groups, future research needs to measure communication visibility to replicate and strengthen our findings.

Second, our study only examined trust in coworkers as a mediator between communication visibility and helping behavior from the perspective of employee cognition. Future studies could explore the mechanisms from compound perspectives. For instance, the perspective of employee emotion could be added. Moreover, we only explored the proactive personality as a character trait in our model. Leadership characteristics, job characteristics, or organizational rules for regulating visible information should also be considered. Future research could explore additional factors that exert different effects on the associations between communication visibility and employee behavior.

Third, we only collected data from one enterprise. Given that this enterprise was ready to use ESM and cultivated open discussions among employees, our findings may have been influenced by enterprise culture. Previous studies found that organizational culture could influence the use and introduction of digital tools [54]. Thus, future studies should consider the role of enterprise culture. Allowing for the positive role of supervisor support [55] and organizational justice [56] in the organization, we assume that our model could also be adapted for organizations with fair and supportive cultures. Future studies could attempt to replicate our research in different cultures.

## 6. Conclusions

Based on communication visibility theory and social cognitive theory, this study highlights the underlying psychological mechanisms through how communication visibility enhances employee helping behavior. Following an analysis of data gathered from 149 members of a company in China, the results confirm a positive relationship between communication visibility and helping behavior. Specifically, communication visibility facilitates employees knowing more about their coworkers, which in turn helps to build trust in coworkers. In addition, the extent to which communication visibility is translated into helping behavior via trust in coworkers is moderated by the level of proactive personality. That is, communication visibility may enhance helping behavior more for employees with a highly proactive personality. Future research could explore more ways and boundaries to enhance employee helping behavior from the perspective of communication visibility theory.

## Figures and Tables

**Table 1:** Demographic characteristics of participants.

Group	Sample Size	Department	Job Title	Job Type	Characteristics of Enterprise WeChat Usage
Control group	55	Security department/ Customer service department	Receptionist/ Service staff/ Legal personnel…	Full time/ Part time	Old version: for simple collaboration, such as sending files to coworkers, but invisible to a third party
Treatment group	94	Human resource department/ Operating department/ Finance department/ Administrative department/ Marketing department	Human resources specialist/ Accounting staff/ Administrative clerk/ Sales staff/…	Full time/ Part time	New version: free posting and communication with anyone in the organization; visible to a third party

**Table 2:** Means, standard deviations, and correlations among variables.

Variable	M	SD	1	2	3	4	5	6	7
1. Gender	0.44	0.50							
2. Age	2.08	0.63	−0.33 **						
3. Education	2.56	0.68	0.26 **	−0.37 **					
4. Work tenure	2.80	0.99	−0.34 **	0.42 **	−0.30 **				
5. Communication visibility	0.63	0.48	0.02	0.03	−0.05	0.03			
6. Trust in coworkers	4.06	0.43	−0.14	−0.01	−0.01	−0.01	0.25 **		
7. Helping behavior	4.33	0.54	−0.04	−0.09	0.17 *	−0.03	0.16 *	0.21 **	
8. Proactive personality	3.64	0.52	−0.21 *	0.14	0.002	0.20 *	0.23 **	0.29 **	0.32 **

Note. *N* = 149. * *p* < 0.05, ** *p* < 0.01 (two-tailed). Communication visibility is a binary variable.

**Table 3:** Model fits of measurement models.

Models	χ^2^	df	χ^2^/df	CFI	TLI	RMSEA
Three-factor model ^a^	356.15	213	1.67	0.92	0.90	0.07
Two-factor model ^b^	911.41	229	3.98	0.59	0.55	0.14
One-factor model ^c^	1209.84	230	5.26	0.42	0.36	0.17

Note. a: In this model, all items were influenced by their own factors respectively; b: In this model, items for trust in coworkers and helping behavior were influenced by the same factor, items for other variables were influenced by their own factors respectively; c: In this model, there was only one factor influencing all variables.

**Table 4:** Regression results.

Variables	Trust in Coworkers		Helping Behavior					
	Model 1	Model 2	Model 3	Model 4	Model 5	Model 6	Model 7	Model 8
Gender	−0.14	−0.14	−0.11	−0.11	−0.08	−0.08	−0.04	−0.01
Age	−0.03	−0.04	−0.06	−0.06	−0.05	−0.05	−0.07	−0.05
Education	0.001	0.01	0.14 *	0.15 *	0.15 *	0.14 *	0.12	0.11
Work tenure	−0.02	−0.02	0.01	0.01	0.01	0.02	−0.01	0.001
Communication visibility		0.22 ***		0.19 *	0.14			
Trust in coworkers					0.22 *	0.26 **	0.16	0.11
Proactive personality							0.30 ***	0.31 ***
Proactive personality * Trust in coworkers								0.37 *
R2	0.02	0.08	0.04	0.07	0.10	0.08	0.15	0.18
△R2	0.02	0.06 ***	0.04	0.03 *	0.03 *	0.04 **	0.07 ***	0.03 *

Note. *N* = 149. * *p* < 0.05, ** *p* < 0.01, *** *p* < 0.001 (two-tailed).

**Table 5:** Conditional indirect effects.

Moderator	Level	Effect	SE	95% CI
Proactive personality	Low	−0.02	0.05	[−0.12, 0.06]
	High	0.07	0.03	[0.01, 0.15]
	Index of moderated mediation	0.09	0.06	[0.008, 0.228]
